# A Practical Treatise on Impotence, Sterility and Allied Disorders of the Male Sexual Organs

**Published:** 1888-04

**Authors:** 


A PRACTICAL TREATISE ON IMPOTENCE, STERILITY AND ALLIED DISORDERS OF THE MALE SEXUAL ORGANS. By Samuel W. Gross, A. M., M. D., LL. D., Professor of Surgery in the Jefferson Medical College, etc. Third edition. Philadelphia: Lea Brothers & Company.

This now classical work on the subject of impotence and sterility in the male needs no extended review, for it is already well known to the medical profession. Dr. Gross has by his tireless labor done more towards clearing up the diagnosis and treatment of these obscure cases than any other American physician. The fact that this book has rapidly run through two large editions, and the author is now forced to issue a third, is good and sufficient evidence of its excellence.

